# Weight loss and dysgeusia in relapsed/refractory multiple myeloma patients treated with talquetamab

**DOI:** 10.1002/jha2.971

**Published:** 2024-07-03

**Authors:** Syed Naqvi, Asis Shrestha, Marah Alzubi, Jawad Alrawabdeh, Sharmilan Thanendrarajan, Maurizio Zangari, Frits van Rhee, Carolina Schinke, Samer Al Hadidi

**Affiliations:** ^1^ Myeloma Center University of Arkansas for Medical Sciences Little Rock Arkansas USA; ^2^ Department of Internal Medicine University of Jordan Amman Jordan

**Keywords:** dysgeusia, multiple myeloma, talquetamab, weight loss

## Abstract

Talquetamab is an approved therapy for relapsed multiple myeloma. This study examined dysgeusia and weight loss occurrences, alongside investigating symptom reversibility post‐treatment cessation. Dysgeusia was prevalent, persisting in 15% of patients. On average, patients lost 6% of their weight during treatment, with weight loss persisting in about half of the patients post‐discontinuation. Weight loss and dysgeusia are important adverse events to consider while on talquetamab treatment. Extending dose intervals can potentially prevent such adverse events and should be studied in future prospective clinical trials.

1

Talquetamab, an approved bispecific monoclonal antibody, binds to both the G‐protein coupled receptor family C group 5 member D (GPRC5D) receptor on myeloma cells and the CD3 receptor on T cells, offering a targeted therapy option for patients with relapsed refractory multiple myeloma (RRMM). It has demonstrated high response rates in heavily pretreated RRMM patients [1, 2, 3]. The expression of GPRC5D extends to both malignant and non‐malignant plasma cells, as well as healthy tissues like the epithelial cells of the tongue [2, 4]. Patients undergoing treatment with talquetamab commonly experience skin and nail complications, along with taste disturbances, possibly linked to the expression of GPRC5D in hard keratin‐producing cells. This localized expression likely explains the potential mechanism behind dysgeusia observed with talquetamab administration. Limited information exists regarding the extent of weight loss and alterations in taste associated with the administration of talquetamab. In the MonumentaL‐1 study, 30% of patients have decreased weight and 63% of patients have dysgeusia with subcutaneous talquetamab and all of the events were of low grade [4]. In addition, it is not well understood whether these side effects are reversible upon discontinuation of treatment. The objective of this study was to assess the occurrence of dysgeusia and weight loss in patients undergoing treatment with talquetamab, as well as to investigate the reversibility of these symptoms after treatment cessation.

The study involved patients diagnosed with RRMM who received at least one dose of talquetamab at the University of Arkansas for Medical Sciences until December 2022. Data collected retrospectively included patient demographics, baseline disease characteristics, prior therapies, number of talquetamab doses administered, and adverse events (AEs) such as weight loss, dysgeusia, cytokine release syndrome (CRS), immune effector cell‐associated neurotoxicity syndrome (ICANS), and other side effects. AEs were graded using the Common Terminology Criteria for Adverse Events (CTCAE), with dysgeusia categorized into grades 1 and 2. Additionally, a Body Mass Index‐Adjusted Weight Loss Grading System (BMI‐WLGS) was employed to monitor weight loss trends during treatment [5]. Percentage weight loss (%WL) was calculated using the formula [weight lost (kg)/current weight (kg) × 100], providing a standardized method for assessing weight loss trends relative to the individual's BMI.

Seventeen patients were included in our analysis. Baseline characteristics are shown in Table 1. Among these patients, nine patients (53%) were male. Ten patients (59%) were Black. The median age at diagnosis was 62 years (range: 51–75, interquartile range [IQR] 9). Six patients (35%) had Eastern Cooperative Oncology Group (ECOG) performance status ≥2. The median number of prior lines of therapy was seven (range: 3–12, IQR 4). Fourteen patients (82%) received at least one prior autologous hematopoietic stem cell transplant. Nine patients (52%) had extramedullary disease. The median number of doses received was 14.8 (range: 2–29, IQR 14). Patients received either 0.4 mg/kg weekly or 0.8 mg/kg every other week. The median time on treatment was 4.36 months (range: 1.2–15.8 months, IQR 6.73). The overall response rate (ORR) was 76%. CRS occurred in 12 patients (70.6%), with five patients (29.4%) experiencing grade II or higher CRS. ICANS occurred in four patients (23.6%), and two patients (11.8%) had grade II ICANS.

**TABLE 1:** Patient baseline characteristics at the initiation of talquetamab.

Characteristic	Frequency
Age at diagnosis: years, median (min‐max)	62 (51–75)
Gender: Male, *n* (%)	9 (52.9)
Race: Black, *n* (%)	10 (59)
White, *n* (%)	7 (41.2)
R‐ISS ≥ 2 at the time of diagnosis, *n* (%)	10 (83.3)
Myeloma subtype: IgG, *n* (%)	10 (58.8)
Light Chain Subtype: Kappa, *n* (%)	9 (52.9)
ECOG performance status: ≥2, *n* (%)	6 (35)
Median LOT^*^, n (min‐max)	7 (3–12)
Prior 1 ASCT^**^	7 (41.2)
Prior 2 ASCT	3 (17.6)
High risk Cytogenetics^***^, *n* (%)	8 (47)
Extramedullary disease (EMD), *n* (%)	9 (52)
PET scan ≥ 3 focal lesions, *n* (%)	13 (76)

* LOT = Line of therapies.

** ASCT = autologous stem cell transplant.

*** High risk Cytogenetics = per IMWG criteria.

Fourteen patients (82%) developed significant weight loss during treatment with talquetamab. Median BMI before treatment was 27 (range: 19.7–50.4, IQR 6.69). The median weight loss was 11.6 lbs (range: 0–30.40 lbs, IQR 14.4), and the median decrease in BMI was 1.76 (range: 0–5.56, IQR 2.16), Median percentage of weight loss was 6.1% from weight prior to talquetamab (range: 0–14.3%, IQR 4.76%) and one patient had grade IV weight loss per the BMI‐WLGS grading system. Weight loss was calculated using data from all patients, including those who did not lose weight. Fourteen patients (82%) reported dysgeusia during talquetamab treatment (Table 2). Eight of these patients had grade I dysgeusia, and six patients had grade II dysgeusia. Two patients had persistent dysgeusia for more than 4 months even after discontinuation of talquetamab treatment at the time of the last follow‐up. Eight patients had weight gain after talquetamab discontinuation (*n* = 8/14, 57%), with five returning to their baseline weight or higher. Six of these patients had dysgeusia during talquetamab, and all six had resolution of dysgeusia after talquetamab discontinuation. We found no correlation between longer time of therapy and severity of weight loss. The median follow up time while on talquetamab was 6.73 months, ranging from 2.07 to 17.71, IQR 9.82. Median progression‐free survival (PFS) was 6.74 months from initiation of talquetamab. No difference in PFS was found between patients who developed dysgeusia and patients who did not develop dysgeusia.

**TABLE 2:** Dysgeusia and body mass index (BMI)‐adjusted weight loss grading system.

Patient no.	Dysgeusia grade	Persistent dysgeusia after discontinuation of Talquetemab	Other dermatologic and/or gastrointestinal AEs
1	I	No	Rash and pruritus
2	I	No	Nail bed changes
3	II	No	Nail changes
4	I	No	Diarrhea and skin peeling
5	0	N/A	Diarrhea, rash, and skin peeling
6	I	No	Nausea
7	I	No	Dry skin, skin peeling, rash, and brittle fingernails
8	0	N/A	Mouth sores, dry skin, and skin peeling
9	II	No	Skin peeling, diarrhea, and abdominal pain
10	I	No	None
11	I	No	Actinic keratosis, red tongue, and falling‐off fingernails
12	II	No	Dry skin and loss of toenails
13	I	No	Dry mouth, skin peeling, and decreased appetite
14	II	No	Skin peeling and decreased appetite
15	II	Yes	Diarrhea
16	II	Yes	Skin exfoliation and intermittent diarrhea
17	0	N/A	None

Abbreviations: AE, adverse event; N/A, not applicable.

This study demonstrates the prevalence of weight loss and dysgeusia caused by talquetamab use. In prior studies, the most common treatment‐emergent AEs with talquetamab were CRS, cytopenia, and dermatologic toxicities and the majority of them were of low grade [4]. It is important to note that the dysgeusia grading system only has grades I and II.

Our study results showed that more than two‐thirds of patients treated with talquetamab developed BMI‐adjusted WLGS grade ≥II and dysgeusia (Figures S1 and S2). Patients, on average, lost 6% of their initial weight during talquetamab treatment. Weight loss persisted in approximately half of the patients after discontinuation of talquetamab. A recent study assessed the safety and efficacy of reduced‐intensity dosing with talquetamab in patients with RRMM. Patients were enrolled in the MonumenTAL‐1 trial, and those who switched to less frequent or reduced dosing with talquetamab were evaluated. Results showed that most patients who switched to reduced dosing maintained or deepened their responses to talquetamab, and AEs generally improved over time, however, weight loss didn't improve despite less frequent or reduced dosing. These findings suggest that reduced or less frequent dosing of talquetamab may help mitigate AE while sustaining treatment response, warranting further investigation into its clinical implications [6].

Another study aimed to investigate the impact of talquetamab therapy on taste perception, salivary flow, dry mouth sensation, and quality of life in patients with RRMM. Using pre‐ and post‐treatment assessments, eight RRMM patients were evaluated for changes in taste perception, salivary flow rates, dry mouth sensation, and overall quality of life following initiation of talquetamab therapy. The results revealed a rapid and significant decrease in taste perception scores and exacerbated dry mouth sensation in all patients, despite no observed change in salivary flow rates. These oral toxicities were found to significantly impair patients' overall quality of life, highlighting the importance of proactive management strategies to address these adverse effects and optimize treatment outcomes [7].

Our findings underscore the importance of discussing and monitoring weight loss while undergoing talquetamab treatment. Such treatment‐emergent AEs need to be discussed with patients and monitored during treatment. Identifying and addressing weight loss and dysgeusia early while on talquetamab treatment with extended dose intervals can potentially prevent additional complications (including oropharyngeal infections and nutritional deficiencies) and discomfort. Future trials may investigate the role of various supportive measures, and/or dose modifications/finite time treatment in ameliorating such AEs to ensure patients’ tolerance.

## AUTHOR CONTRIBUTIONS

Samer Al Hadidi conceived the research idea. Syed Naqvi and Asis Shrestha worked on data collection. Samer Al Hadidi and Syed Naqvi wrote the initial draft. All authors critically reviewed and approved the final submission of the manuscript.

## CONFLICT OF INTEREST STATEMENT

Samer Al Hadidi reports receiving honoraria from Jansen, Sanofi, and Pfizer.

## FUNDING INFORMATION

No funding was received for this work.

## ETHICS STATEMENT

The authors have confirmed ethical approval statement is not needed for this submission.

## PATIENT CONSENT STATEMENT

The authors have confirmed patient consent statement is not needed for this submission.

## CLINICAL TRIAL REGISTRATION

The authors have confirmed clinical trial registration is not needed for this submission.

## Supporting information

Supporting Information

Supporting Information

## Data Availability

Data collected are all published.

## References

[1] U.S. FDA approves TALVEY™ (talquetamab‐tgvs), a first‐in‐class bispecific therapy for the treatment of patients with heavily pretreated multiple myeloma. Accessed March 12, 2024. https://www.jnj.com/media‐center/press‐releases/u‐s‐fda‐approves‐talvey‐talquetamab‐tgvs‐a‐first‐in‐class‐bispecific‐therapy‐for‐the‐treatment‐of‐patients‐with‐heavily‐pretreated‐multiple‐myeloma

[2] Elemian S , Al Hadidi S . Targeting GPRC5D in multiple myeloma. Expert Rev Anticancer Ther. 2024;24(5):229–238. 10.1080/14737140.2024.2343114 38607646

[3] Rafae A , van Rhee F , Al Hadidi S . Perspectives on the treatment of multiple myeloma. Oncologist. 2024;29(3):200–212. 10.1093/oncolo/oyad306 37995307 PMC10911930

[4] Chari A , Minnema MC , Berdeja JG , Oriol A , van de Donk NWCJ , Rodríguez‐Otero P , et al. Talquetamab, a T‐cell‐redirecting GPRC5D bispecific antibody for multiple myeloma. N Engl J Med. 2022;387(24):2232–2244. 36507686 10.1056/NEJMoa2204591

[5] Martin L , Senesse P , Gioulbasanis I , Antoun S , Bozzetti F , Deans C , et al. Diagnostic criteria for the classification of cancer‐associated weight loss. [published correction appears in J Clin Oncol. 2015 Mar 1;33(7):814]. J Clin Oncol. 2015;33(1):90–99. 10.1200/JCO.2014.56.1894 25422490

[6] Fleischer A , Roll M , Panther F , Gelbrich G , Reinhardt F , Riedhammer C , et al. Taste abnormalities emerging during anti‐myeloma therapies including GPRC5D x CD3 bispecific antibody talquetamab. Blood. 2023;142(Supplement 1): 2403. 10.1182/blood-2023-186663

[7] Laheij AMGA , van de Donk NWCJ . Characterization of dysgeusia and xerostomia in patients with multiple myeloma treated with the T‐cell redirecting GPRC5D bispecific antibody talquetamab. Support Care Cancer. 2023;32(1):20. Published 2023 Dec 14. 10.1007/s00520-023-08233-0 38092979

