# Correction to “Weight Loss and Dysgeusia in Relapsed/Refractory Multiple Myeloma Patients Treated With Talquetamab”

**DOI:** 10.1002/jha2.70052

**Published:** 2025-05-19

Naqvi S, Shrestha A, Alzubi M, Alrawabdeh J, Thanendrarajan S, Zangari M, et al. Weight loss and dysgeusia in relapsed/refractory multiple myeloma patients treated with talquetamab. eJHaem. 2024;5:789–792. doi: 10.1002/jha2.971

Carolina Schinke has mistakenly not declared her Conflict of Interest and would like to add a sentence stating “CS has participated in Advisory Board Meetings of Janssen, Pfizer and Arcellx”.

We apologize for this error.

