# Supplementary material for: Weight loss and dysgeusia in relapsed/refractory multiple myeloma patients treated with talquetamab
Source: EJHaem. 2024 Jul 3;5(4):789–92. doi: 10.1002/jha2.971 (PMC11327703; doi:10.1002/jha2.971)
Supplement: Supplementary file 1 — Supporting Information [file JHA2-5-789-s002.docx]

**Supplementary Figure legends**

**Supplemental Figure.1:** Dysgeusia during Talquetamab and its association with weight loss

**Supplemental Figure 2:** Dygeusia after Talquetamab treatment and its association with weight loss

**Supplemental Figure.1:** Dysgeusia during Talquetamab and its association with weight loss

**
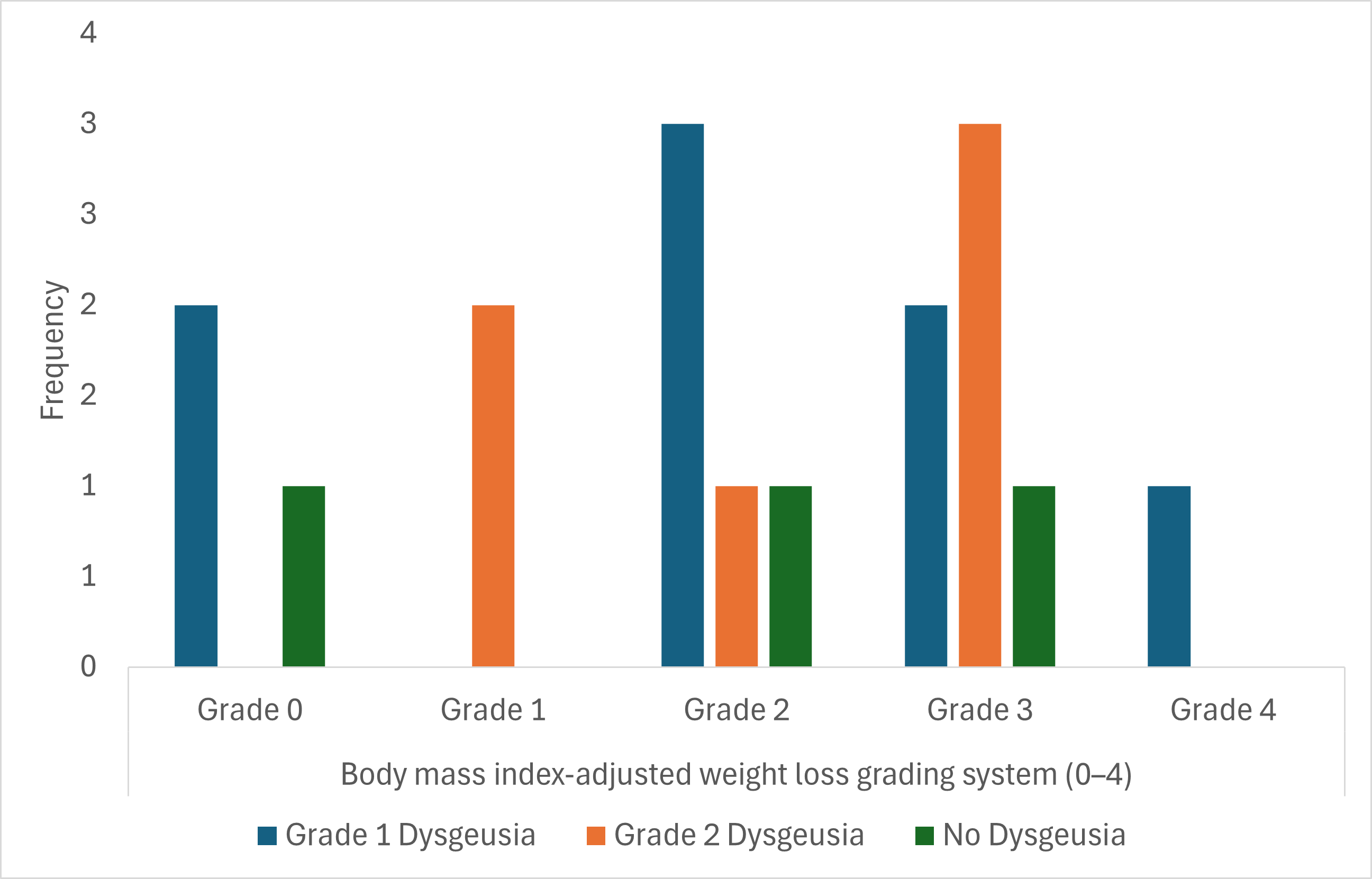
**

**Supplemental Figure 2:** Dygeusia after Talquetamab treatment and its association with weight loss

**
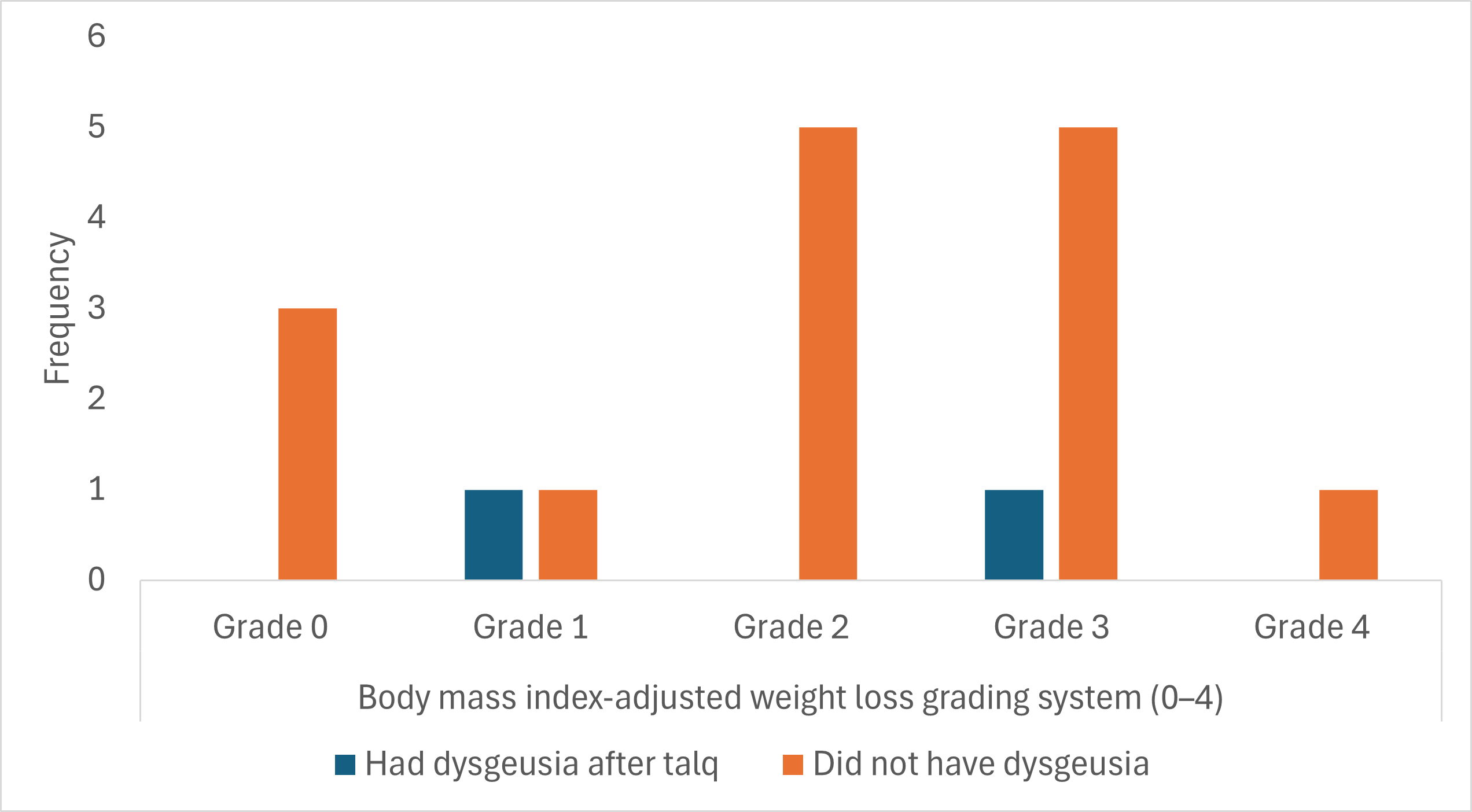
**
