# Supplementary material for: Weight loss and dysgeusia in relapsed/refractory multiple myeloma patients treated with talquetamab
Source: EJHaem. 2024 Jul 3;5(4):789–92. doi: 10.1002/jha2.971 (PMC11327703; doi:10.1002/jha2.971)
Supplement: Supplementary file 2 — Supporting Information [file JHA2-5-789-s001.docx]

**Tweetable Summary Label**

New study reveals significant dysgeusia & weight loss in RRMM patients treated with talquetamab.#mmsm #qualityoflife

**Twitter Handle(s) (up to 7 authors)**

@HadidiSamer @UAMSMyeloma
